# A Common Copy Number Variation (CNV) Polymorphism in the *CNTNAP4* Gene: Association with Aging in Females

**DOI:** 10.1371/journal.pone.0079790

**Published:** 2013-11-06

**Authors:** Leonid Iakoubov, Malgorzata Mossakowska, Malgorzata Szwed, Zhibing Duan, Federico Sesti, Monika Puzianowska-Kuznicka

**Affiliations:** 1 GeneCona, LLC, Vallejo, California, United States of America; 2 PolSenior Project, International Institute of Molecular and Cell Biology, Warsaw, Poland; 3 Department of Human Epigenetics, Mossakowski Medical Research Centre, Warsaw, Poland; 4 Department of Neuroscience and Cell Biology, Robert Wood Johnson Medical School, Rutgers University, New Brunswick, New Jersey, United States of America; 5 Department of Geriatrics and Gerontology, Medical Center of Postgraduate Education, Warsaw, Poland; Yale School of Public Health, United States of America

## Abstract

**Background:**

Aging is a biological process strongly determined by genetics. However, only a few single nucleotide polymorphisms (SNPs) have been reported to be consistently associated with aging. While investigating whether copy number variations (CNVs) could fill this gap, we focused on CNVs that have not been studied in previous SNP-based searches *via* tagging SNPs.

**Methods:**

TaqMan qPCR assays were developed to quantify 20 common CNVs in 222 senior American Caucasians in order to reveal possible association with longevity. The replication study was comprised of 1283 community-dwelling senior European Caucasians. Replicated CNVs were further investigated for association with healthy aging and aging-related diseases, while association with longevity was additionally tested in *Caenorhabditis elegans*.

**Results:**

In the discovery study of ≥80 *vs*.<80 years old seniors, a homozygous intronic CNV deletion in the *CNTNAP4* gene was inversely associated with survival to the age of 80 (OR=0.51, 95%CI 0.29-0.87, p=0.015 before correction for multiple testing). After stratification by sex, association remained significant in females (OR=0.41, 95%CI 0.21-0.77, p=0.007), but not in males (OR=0.97, 95%CI 0.33-2.79, p=1). The finding was validated in a replication study (OR=0.66, 95%CI 0.48-0.90, p=0.011 for females). *CNTNAP4* association with longevity was supported by a marked 25% lifespan change in *C. elegans* after knocking down the ortholog gene. An inverse association of the CNV del/del variant with female healthy aging was observed (OR=0.39, 95%CI 0.19-0.76, p=0.006). A corresponding positive association with aging-related diseases was revealed for cognitive impairment (OR=2.17, 95%CI 1.11-4.22, p=0.024) and, in independent studies, for Alzheimer’s (OR=4.07, 95%CI 1.17-14.14, p=0.036) and Parkinson’s (OR=1.59, 95%CI 1.03-2.42, p=0.041) diseases.

**Conclusion:**

This is the first demonstration for association of the *CNTNAP4* gene and one of its intronic CNV polymorphisms with aging. Association with particular aging-related diseases awaits replication and independent validation.

## Introduction

 Aging is a process that includes various phenotypes, such as longevity, healthy aging, and aging-related diseases. While longevity is a phenotype with moderate heritability [1], other phenotypes of aging, specifically some aging-related diseases like late onset Alzheimer’s disease (LOAD), are more genetically determined, up to 70-80% [2]. Although numerous single nucleotide polymorphisms (SNP) have been reported to be associated with longevity and aging-related diseases [3,4], just a few of them have been consistently replicated [5]. Hence, much of aging-related heritability is still unknown.

Copy number variations (CNVs) have been recently discovered as a new class of polymorphisms. They are defined as DNA segments ranging in size from one kilobase to several megabases that differ among individuals due to deletion, insertion, inversion, duplication or complex recombination [6]. It was hoped that CNVs would help to explain the missing heritability in many genetically determined traits and aging. Indeed, as a result of massive genome-wide associated studies (GWAS), several CNVs have been reported to be associated with aging [7] and complex diseases such as schizophrenia [8] and cancers [9,10]. Nevertheless, the number of CNVs consistently associated with age-related phenotypes seems to be far below initial expectations. 

It appears that a substantial number of CNVs are tagged by SNPs that were well represented in GWAS arrays. Hence, these CNVs have been already indirectly explored through previous SNP studies [11]. This could be one of important reasons why not many of new associations with complex traits have been identified in CNV-based GWAS [12]. In order to focus our effort on CNVs that have not been intensively investigated thus far, we created a list of approximately 200 common CNVs whose tagging SNPs were underrepresented in most SNP-based arrays. 

In the study reported herein, we have randomly selected 20 (Table S1) of these 200 CNVs and analyzed them for associations with longevity. This was followed by the analysis of associated CNVs with healthy aging and aging-related conditions and diseases including cognitive impairment, LOAD, and Parkinson’s disease (PD). Since aging is a process that differs substantially between men and women [13], after analyzing the data for men and women combined, an additional analysis was performed for each sex group separately in order not to miss sex-specific associations.

## Materials and Methods

### Genomic DNA

DNA samples for the discovery study were purchased from Coriell Institute (Camden, NJ) and PrecisionMed Laboratories (San Diego, CA). The samples originated from neurologically normal North American Caucasians over 50 years old. DNA samples from community-dwelling Eastern European Caucasians for the replication study were from the PolSenior collection (Warsaw, Poland). The demography is described in detail below. DNA samples from patients with Alzheimer’s disease and age-matched normal controls originated from North American Caucasians and were purchased from the National Cell Repository for Alzheimer’s Disease (Indianapolis, IN). DNA samples from Parkinson’s disease patients and age-matched normal controls also originated from North American Caucasians, and were obtained from the National Institute of Neurological Disorders and Stroke (NINDS) repository through Coriell Institute. Data regarding the number of samples and mean ages of sample donors are provided in the notes below each table (“Results” section). Control male and female DNA and DNA samples from cell lines used in the model experiment (Figure S1) were purchased from Coriell Institute. 

### PolSenior Collection

 The results obtained in DNA samples from the PolSenior collection (PolSenior study is coordinated by the International Institute of Molecular and Cell Biology in Warsaw, Poland) [14] represent the major evidence basis in this investigation. 

 All PolSenior participants gave a written informed consent for participation in the study. The study protocol was approved by the Bioethical Committee of the Medical University of Warsaw. All data on PolSenior samples, as well as all other data of this investigation were analyzed anonymously.

 For the longevity study, the difference in frequencies of CNV polymorphisms was investigated between two age groups, one consisting of subjects between 65 and 75 years old (mean age 69.9±3.07; n=645), and the other between 80 and 90 years old (mean age 84.5±3.81; n=638). For the study of association with aging-related diseases, of the 1283 PolSenior DNA samples available at the beginning of the current study, complete medical records were available for 1118 individuals (587 males and 531 females). Medical records included data on cardiovascular and respiratory diseases, cancer, diabetes, cognitive impairment and stroke (Table S2).

 In a stratified analysis, healthy participants were identified as those who scored ≥24 points (maximum score of 30 points) on the Polish version of the Mini-Mental State Examination (MMSE) test and did not have any records of cardiovascular diseases, cancer, diabetes, stroke or chronic lung disease. Participants with cognitive impairment were identified as those with MMSE <24 and with or without any of above-mentioned chronic diseases. Participants with “other pathologies” had an MMSE score of ≥24 and at least one of the above-mentioned chronic diseases in their medical records.

### Selection of CNV variants

In order to investigate CNVs that have not been intensively studied in GWAS thus far, we created a list of about 200 CNVs that were not represented in most of the SNP-based arrays: AFFY 500K (Affymetrix, Santa Clara, CA) and ILMN HumanHap 300, 500, 650Y (Illumina, San Diego, CA) by their tagging SNPs. The CNVs were selected from the plurality of more than 30,000 CNVs that are catalogued in the Database for Genomic Variants (http://projects.tcag.ca/variation/?source=hg18). The absence of tagging SNPs in the content of indicated arrays was monitored using “SNP array” tracks of the site. Most of the selected CNVs were common bi-allelic CNVs with intronic location. The 20 CNVs reported here in Table S1 were randomly selected from the list for testing in the discovery study. The only selection criterion was the minor allele frequency of >10%. This was needed to increase the study power, since the number of samples available for the initial testing was limited. The IDs were either from the recently released reference catalog of HapMap CNVs [15], the Database for Genomic Variants, or NCBI dbVar database.

### CNV genotyping

For accurate quantification of each CNV, we have developed a corresponding TaqMan real time qPCR assay based on the delta-delta Ct method (without standard curve) using a 384-well platform of Lightcycler 480 (Roche Diagnostics Corporation, Indianapolis, IN). The actual quantification of a particular sample was achieved after normalization of a raw Ct value for this sample in accordance with median Ct value for established reference controls targeted to non-variable gene loci of sex chromosomes. Primer-probe sequences for reference controls and for the quantification of CNVR6782.1 in the *CNTNAP4* gene are provided in Table S3. All primers and probes were custom ordered from Biosearch Technologies, Inc. (Novato, CA). Each PCR sample contained 5 μl of master mix (Roche Diagnostics Corporation, Indianapolis, IN), 2 μl of primer-probe mixture (final concentration of 900 nM for each primer and 250 nM for the probe) and 5 ng of the DNA in 3 μl of PCR-grade water. The amplification conditions were: 5 min at 95°C and 40 cycles of 95°C for 15 sec and 60°C for 1 min. In a model experiment, the method was shown to accurately quantify targets that vary in the range from 0 to 5 copies per genome (Figure S1). 

### *C. elegans* longevity model

The detailed procedures were previously described [16,17]. Briefly, nematodes were grown in standard 10 cm NGM plates + OP50 *E. coli* until a large population of gravid adults was reached (3-5 days). The animals were collected in 50 ml Falcon tubes, washed in M9 buffer (22 mM KH_2_PO_4_, 22 mM NaH_2_PO_4_, 85 mM NaCl, 1 mM MgSO_4_), and treated with 10 volumes of basic hypochlorite solution (0.25 M NaOH, 1% hypochlorite) freshly mixed. Worms were incubated at room temperature for 10 min, the eggs (and carcasses) collected by centrifugation at 400x g for 5 min at 4°C, incubated overnight in M9 buffer and seeded on standard NGM plates. Four-day-old worms were transferred on NGM plates supplemented with 25 mg/ml carbenicillin and 1 mM IPTG, then seeded with HT115 *E. coli* containing an empty control vector (L4440) or expressing double-stranded *NRX-1* RNAi. Worms were examined every day until death and were scored as dead when they were no longer able to move even in response to prodding with a platinum pick. Each day, worms were transferred to a fresh plate containing bacteria. Each single experiment started with 30 worms per plate.

### Statistical analysis

We have used Fisher’s exact test and 2x2 contingency tables to assess association with age and age-related diseases in various populations. Two-sided P values of <0.05 were considered statistically significant. In tandem with Fisher's exact test, odds ratios and 95% confidence intervals for the odds ratio were calculated. Differences in the length of life of *C. elegans* were evaluated by Student’s t-test.

## Results

### Association of the CNVR6782.1del/del variant in the *CNTNAP4* gene with longevity

 Of twenty CNVs investigated, only common bi-allelic CNV, R6782.1 located in one of the 3’ introns of the *CNTNAP4* gene from the neurexin superfamily, was significantly associated with longevity. It was an inverse association for the CNVR6782.1del/del variant (OR=0.51, p=0.015 before Bonferroni correction for multiple testing) (Table 1). This association has been tested as a pre-specified hypothesis in the replication study of 1283 community-dwelling Caucasians from Eastern Europe. Consistent with the discovery study, in the replication study, the del/del variant was less frequent in the 80-90 age group than in the control 65-75 years old group, showing an inverse association with survival to the age of 80 years and over (OR=0.78, p=0.028) (Table 1).

**Table 1:** Prevalence of the *CNTNAP4* CNVR6782.1del/del variant in populations and sub-populations of discovery and replication studies in aging.

Sex	Study	N	Prevalence (%)				
			age<80*	age≥80*	OR	95%CI	Fisher p
M+F	Discovery	222	57	40	0.51	0.29-0.87	**0.015**
	Replication	1283	47	41	0.78	0.62-0.96	**0.028**
F	Discovery	162	60	30	0.41	0.21-0.77	**0.007**
	Replication	643	47	37	0.66	0.48-0.9	**0.011**
M	Discovery	60	55	55	0.97	0.33-2.79	1
	Replication	640	47	45	0.9	0.66-1.23	0.579

F: females; M: males.

For the discovery study, the mean age of females was 66.7±10.78 years in the <80 years old (n=89) and 86.4±4.91 years in the ≥80 years old (n=73). In males, it was 63.5±7.76 years (n=38) and 83.9±3.21 years (n=22), respectively. For the replication study, the mean age of females was 69.7±2.89 years in the <80 years old (n=325) and 84.4±4.0 years in the ≥80 years (n=318). In males, it was 70.1±3.28 years (n=320) and 84.7±3.63 years (n=320), respectively.

In each of the two studies, the association of the *CNTNAP4* CNVR6782.1del/del variant with longevity was statistically significant in females, but not in males (females OR=0.41, p=0.007, and males OR=0.97, p=1 in the discovery study; females OR=0.66, p=0.011, and males OR=0.9, p=0.579 in the replication study) (Table 1). 

In A representative sample of PolSenior Collection participants aged <80 years (males and females; n=625), the genotype frequencies for the CNVR6782.1 variants were 47.2% for del/del, 39.5% for in/del and 13.3% for in/in variants, a distribution that did not deviate from Hardy-Weinberg equilibrium (p=0.67).

### Association with longevity for the *C. elegans* ortholog of the human *CNTNAP4* gene

**Figure 1:**
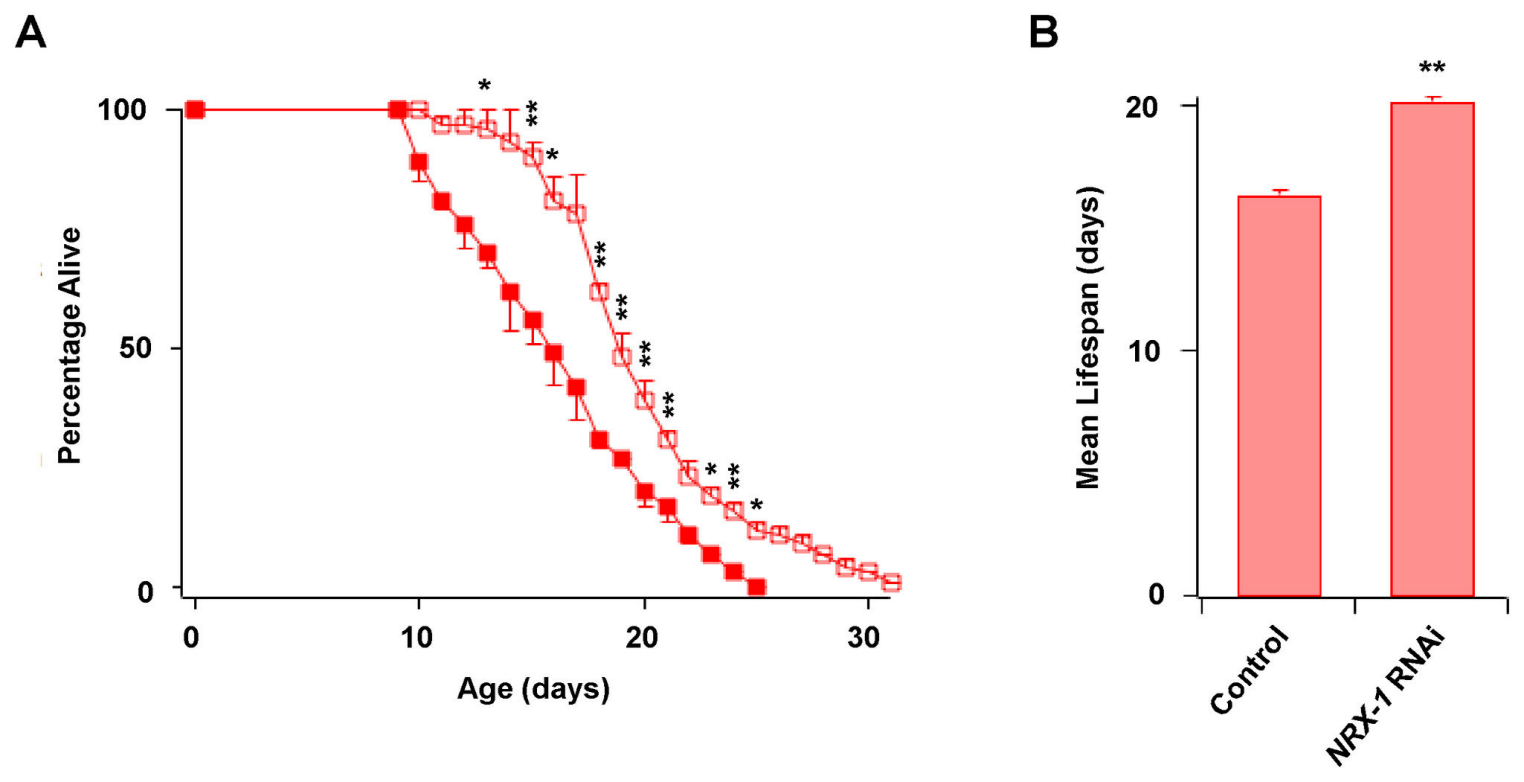
NRX-1 knock down increases longevity in *C. elegans*. A. Lifespan curves for N2 *C. elegans* worms (Bristol strain) in control conditions (filled squares) or subjected to *NRX-1* bacterial RNAi (hollow squares). B. Mean lifespan of control worms and worms subjected to *NRX-1* RNAi in three independent experiments. Statistical significance was evaluated by Student’s t-test using Excell^®^ software. Statistically significant differences from control are indicated by symbols * (p<0.05) and ** (p<0.01).

Neurexins are implicated in autistic spectrum disorder, schizophrenia, drug addiction and angiogenesis [18]. Based on studies of ortholog genes in *C. elegans* aging models, it has been suggested that some neurexins also contribute to the process of aging [19]. Here, we investigated the *NRX-1* gene, the *C. elegans* ortholog of the mammalian *CNTNAP4* gene [20]. Lifespan curves for control worms and worms subjected to *NRX-1* knock down by bacterial RNAi are shown in Figure 1A. Inactivation of *NRX-1* prolonged the maximal lifespan by up to six days. The increase in mean lifespan went up from 16.4±0.19 days in the control to 20.2±0.19 days in *NRX-1* RNAi-treated worms (Figure 1B), a significant 25% increase. 

### Association of the CNTNAP4 R.6782.1del/del variant with healthy aging and age-related diseases

 A strategy for further investigation was based on the concept by Perls et al. [21] predicting an age-related dropout within a population for these genotypes that are linked to aging-related deadly diseases. According to the concept, the genotype that is inversely associated with longevity is expected to be positively associated with at least some of such diseases. We tested this in the group of community-dwelling senior females from the replication study, where full medical records were available. After removing participants with chronic diseases, the analysis revealed that the inverse association of the *CNTNAP4* R6782.1del/del variant with healthy aging was statistically significant in women and had even greater effect size than the association for longevity: OR=0.39, 95%CI 0.19-0.76, p=0.006 (Table 2) *vs.* OR=0.66, 95%CI 0.48-0.90, p=0.011 (Table 1). No significant association of the variant with healthy aging was observed in men. As shown below and consistent with the above-mentioned hypothesis of Perls et al., the same del/del variant that was inversely associated with female longevity and healthy aging, demonstrated a significant positive association with some age-related pathologies in females. Taking into account that the *CNTNAP4* gene is mainly expressed in the central nervous system (see the “Discussion” section), we concentrated on its disorders. Our initial computation has revealed a statistically significant positive association of the del/del variant with cognitive impairment in PolSenior study participants, OR 2.17 and p=0.024 (Table 3), while no statistically significant association was observed for other diseases that are listed in the Table S2. This result prompted us to test the possibility of a positive association of the del/del variant with some age-related diseases that often have a cognitive impairment component in their pathogenesis, such as Alzheimer’s and Parkinson’s diseases. In independent case-control studies using appropriate DNA collections, we observed a positive association of the *CNTNAP4* R6782.1del/del variant with both diseases. For the association with Alzheimer’s disease, the OR was 4.07, p=0.036 (Table 4), and for Parkinson’s disease, the OR was 1.59, p=0.041 (Table 5).

**Table 2:** Prevalence of the *CNTNAP4* CNVR6782.1del/del variant in relation to healthy aging (PolSenior study).

Sex	n	Prevalence (%)						
		age<80*	age≥80*	OR	95%CI		Fisher p	
M+F	362	50	39	0.65	0.41-0.99		**0.049**	
F	167	52	30	0.39	0.19-0.76		**0.006**	
M	195	47	46	0.95	0.53-1.70		0.884	

F: females; M: males.

The mean age of healthy females was 69.3±2.76 years in the <80 years old sub-group (n=107) and 83.5±3.63 years in the ≥80 years old subgroup (n=60). In males it was 70.0±3.05 years (n=121) and 84.9±3.57 years (n=74), respectively.

**Table 3:** Prevalence of the *CNTNAP4* CNVR6782.1del/del variant in ≥80 years old seniors (PolSenior collection) with cognitive impairment, and with other pathologies in the absence of cognitive impairment.

Disease	Sex	n	Prevalence (%)				
			Control*	Case**	OR	95%CI	Fisher p
Cognitive impairment	M+F	341	39	49	1.53	0.98-2.38	0.060
	F	172	30	48	2.17	1.11-4.22	**0.024**
	M	169	46	51	1.20	0.65-2.20	0.642
Other pathologies	M+F	316	39	41	1.08	0.68-1.70	0.817
	F	140	30	34	1.19	0.57-2.44	0.716
	M	176	46	38	1.01	0.55-1.83	1.0

M: males; F: females.

The mean age was 83.5±3.63 years (n=60) in healthy females, 85.2±3.84 years in females with cognitive impairment (n=112), and 83.1±4.36 in females with other pathologies (n=80). In males, it was 84.9±3.57 years (n=74), 85.5±3.30 (n=95) and 84.0±3.23 (n=102), respectively.

* Subjects with MMSE score ≥24 and no chronic diseases in medical records.

** In “Cognitive impairment” subgroup, these are subjects with MMSE score <24 and other pathologic conditions might or might not be present. In “Other pathologies” subgroup, these are subjects with MMSE score ≥24, but with one or more of chronic diseases present.

**Table 4:** Prevalence of the *CNTNAP4* CNVR6782.1del/del variant in late-onset Alzheimer’s disease.

		Prevalence (%)				
Sex	n	Healthy	Alzheimer's	OR	95%CI	Fisher p
F+M	111	38	49	1.61	0.75-3.42	0.253
F	46	30	63	4.07	1.17-14.14	**0.036**
M	65	45	42	0.88	0.32-2.35	0.807

F: females; M: males.

The mean age was 80.7±3.41 years in healthy females (n=27) and 80.7±5.34 years in females with LOAD (n=19). In males, it was 77.5±5.46 years (n=29) and 78.9±4.01 years (n=36), respectively.

**Table 5:** Prevalence of the *CNTNAP4* CNVR6782.1del/del variant in Parkinson’s disease.

		Prevalence (%)				
Sex	n	Healthy	Parkinson's	OR	95%CI	Fisher p
F+M	655	47.0	49.4	1.17	0.85-1.58	0.348
F	346	45.0	55.0	1.59	1.03-2.42	**0.041**
M	309	49.0	43.4	0.83	0.53-1.30	0.427

F: females; M: males.

The mean age was 70.8±9.53 years in healthy females (n=177) and 70.8±8.43 years in females with PD (n=169). In males, it was 69.6±9.82 years (n=150) and 70.3±9.15 years (n=159), respectively.

## Discussion

Twenty common CNVs from our list of CNVs that could not be efficiently investigated for the association with various traits in past GWAS were tested for associations with aging phenotypes. An inverse association with longevity was found for a common intronic CNV in the *CNTNAP4* gene. Female carriers of the CNVR6782.1del/del variant of this gene, when compared with non-carriers, demonstrated a statistically significant decrease in the probability of survival to 80 years of age in both the discovery and replication studies, on average by 59% and 34%, respectively. The association of the *CNTNAP4* gene with longevity was validated in our *C. elegans* experiments where knocking down of the *CNTNAP4* ortholog, the *NRX-1* gene, led to a marked 25% lifespan increase. Taken together, these data underscore the role of neurexins in the regulation of longevity. However, particular mechanisms beyond the lifespan extension after the *NRX-1* gene knock-down in *C. elegans* and gender-specific decrease of survival in humans associated with a homozygous deletion of a relatively small piece of intronic DNA from the *CNTNAP4* gene (see Table S1 for the CNVR6782.1 exact size and location) remain to be elucidated in future studies. 

Other important findings of this investigation were the inverse association of the CNVR6782.1del/del variant with healthy aging over the age of 80 and its positive association with several central nervous system aging-related pathologies, such as cognitive impairment, Alzheimer’s, and Parkinson’s diseases. The association with pathologies of this type is concordant with what is known about the *CNTNAP4* gene biology. The encoded CASPR4 protein is a type I transmembrane protein that is mostly expressed in various structures of the central nervous system. Its extracellular region contains a number of well-defined domains (discoidin, laminin G and fibrinogen-like), while its cytoplasmic C-terminal end contains a type II binding site for PDZ domains [22,23]. Notably, contactin-associated protein-like 2 (CASPR2), most closely related to CASPR4, also contains an intracellular PDZ domain binding site and utilizes it while forming complexes with some intermediate proteins. By doing so, it takes part in the spatial organization of potassium channels in Ranvier nodes of myelinated axons [24], the channels being crucial for efficient nerve impulse conduction. Consequently, CASPR2 and the encoding *CNTNAP2* gene, are consistently associated with various nervous system-related disorders [25–29]. Taking into account the predominant localization of CASPR4 in various brain structures and its similarity to CASPR2, we hypothesized that the *CNTNAP4* variants are also associated with nervous system disorders. Indeed, in this study, the association of the *CNTNAP4* CNVR6782.1del/del variant with cognitive impairment, defined as an MMSE score of <24, was found for community-dwelling females ≥80 years old. Since even mild cognitive impairment (scores 21-24) is a risk factor for the LOAD [30], the variant was also tested in this disease and in PD, the second most common neurodegenerative disorder that manifests itself as movement deficits and, in advanced cases, as a cognitive impairment. The fact that the variant appeared to be associated with both LOAD and PD can be considered a validation of its putative role in cognitive impairment. Nevertheless, it should be emphasized here that particular associations with each of the two diseases, LOAD and PD, needs replication and further validation by other investigators. 

The inverse association of the *CNTNAP4* CNVR6782.1del/del variant with longevity and healthy aging on one side, and its positive association with aging-related diseases on the other, is consistent with the concept of demographic selection [21]. The concept predicts the dropout of genotypes that are linked to aging-related deadly diseases due to premature mortality of their carriers as the cohort achieves older age. However, only a limited number of the plurality of genetic markers that are associated with aging-related diseases were found to be additionally associated with longevity and healthy aging [30], even when combinations of disease-associated markers were investigated [31]. Most possibly, the marker’s association with several common diseases is needed in order for the association to be reflected in longevity and healthy aging to the extent that it might be registered experimentally. Importantly, in addition to the association with cognitive impairment, LOAD, and PD (this study), *CNTNAP4* polymorphisms have been shown to be associated with some other phenotypes such as serum level of cholesterol and sphingolipids [32,33], glaucoma [34], autism [35], and D-amphetamine response [36]. In some tumors (breast cancer, adrenocortical adenoma), one of the most frequently deleted regions locates to the 16q23.1 region and overlaps the *CNTNAP4* locus [37,38]. Thus, we speculate that the association of the *CNTNAP4* gene and its CNVR6782.1del/del variant with various diseases in the elderly is a reason for the significant age-associated decrease in the proportion of variant carriers, as presented here, with the lowest frequency in healthy ≥80 years old females. 

 A simultaneous association with several phenotypes of aging, similar to the one described here, has been previously observed for the best-recognized genetic markers in aging, such as *APOE* and *FOXO* polymorphisms. The *APOE* ε4 allele carriers excessively drop out from the stream of aging to 90 years old and over [5], while being overrepresented in aging-related pathologies, such as LOAD and cardiovascular diseases [39,40].  Similarly, the *FOXO1A* and *FOXO3A* variants, in addition to longevity [41], are also associated with diseases such as type 2 diabetes and stroke [42,43]. We speculate that the similarity of the CNVR6782.1del/del variant of the *CNTNAP4* gene to certain *APOE* and *FOXO* polymorphisms with respect to its inverse association with longevity and healthy aging on one side, and positive association with the risk of aging-related diseases on the other, might be an indicator for its good prospects as a prognostic marker in aging. A validation in prospective studies is needed. 

The age groups studied here differ from majority of genetic studies of aging, where genetic profiles of nonagenarians and/or centenarians are compared with genetic profiles of young or elderly adults to search for genes associated with exceptional longevity, as was the case of *APOE* and *FOXO* polymorphisms. Our choice of age groups was based on the fact that starting from the beginning of the past century, the mean lifespan in humans have almost doubled to approximately 80 years in developed countries, and this increase is mostly related to "the rectangularization of the survival curve" [44], not to the progress in exceptional longevity. In our opinion, to achieve further progress in the increase of the mean lifespan, it is important to study the genetics of aging-related diseases in the age interval of 75-80 years, when the current survival curve shows the highest increase in the number of premature deaths [44]. For this reason, we targeted this particular age interval in our replication study, the comparison of prevalent genotypes being made between 65-75 and 80-90 years old age groups. The results indicate a non-proportionally high decrease in the percentage of carriers of the CNVR6782.1del/del genotype in the immediate pre-average age interval of 75-80. One could speculate that therapeutic interventions targeting the *CNTNAP4* gene or related molecules and pathways might be efficient in preventing this dropout in order to further increase the mean lifespan.

The study has a number of limitations. Among them is the relatively small number of subjects in the discovery and Alzheimer’s disease studies. In addition, the discovery study was imbalanced in favor of female vs. male participants. However, the analysis was performed using Fisher exact test that is usually employed to assess statistical significance when sample sizes are small. Additionally, the replication study alleviated the concerns about the suggestive, yet not definite, results in the discovery dataset (uncorrected p value, male/female imbalance). Another serious limitation is the cross-sectional study design. In this regard, a simple comparison of polymorphism frequencies between elderly and younger cohorts is susceptible to artifacts due to population stratification or admixture, birth cohort differences, and secular trends. However, the fact that the observed changes were consistently characteristic for one sex only, allows us to believe that the changes are real events that take place in the aging population and are not a reflection of the difference in genetic background that occurs in every consecutive generation as a result of a non-stop selection, migration, etc. Validation studies in a prospective setting are warranted; however, on the other hand, the strength of this investigation is that the pattern of association of the analyzed genetic variant with phenotypes under study was consistent in terms of both female specificity and effect direction in all four independent case-control studies.

In conclusion, we have demonstrated associations of the *CNTNAP4* gene and its CNVR6782.1del/del polymorphic variant with longevity, healthy aging, and aging-related pathologies such as cognitive impairment and, tentatively, Alzheimer’s and Parkinson’s diseases. Additionally, experiments in *C. elegans* showed the evolutionarily conserved nature of the association with longevity. 

## Supporting Information

Figure S1 **Model TaqMan qPCR assay.** Conditions for TaqMan qPCR were optimized to precisely and reproducibly detect copy numbers from zero to five in control DNA samples with known copy numbers using genetic markers located on ChrX and ChrY and DNA from individuals or cell lines with various known numbers of chromosomes X and Y. X: one copy of chromosome X, Y: one copy of chromosome Y, FoxP3: gene present at chromosome X, G6PD: gene present at chromosome X, SRY: gene present at chromosome Y.
(TIF)

Table S1 **Investigated copy number variants and their harboring genes.** Coordinates are provided for the assembly version NCBI Build 36.3 (UCSC hg 18).
(DOC)

Table S2 **Basic health characteristics of seniors from PolSenior collection.**
(DOC)

Table S3 **Sequences for primers/probes used in the experimental quantification of the CNVR6782.1 in the *CNTNAP4* gene by TaqMan qPCR assay.**
(DOC)
