# Supplementary figures and images for: A Common Copy Number Variation (CNV) Polymorphism in the CNTNAP4 Gene: Association with Aging in Females
Source: PLoS One. 2013 Nov 6;8(11):e79790. doi: 10.1371/journal.pone.0079790 (PMC3819343; doi:10.1371/journal.pone.0079790)

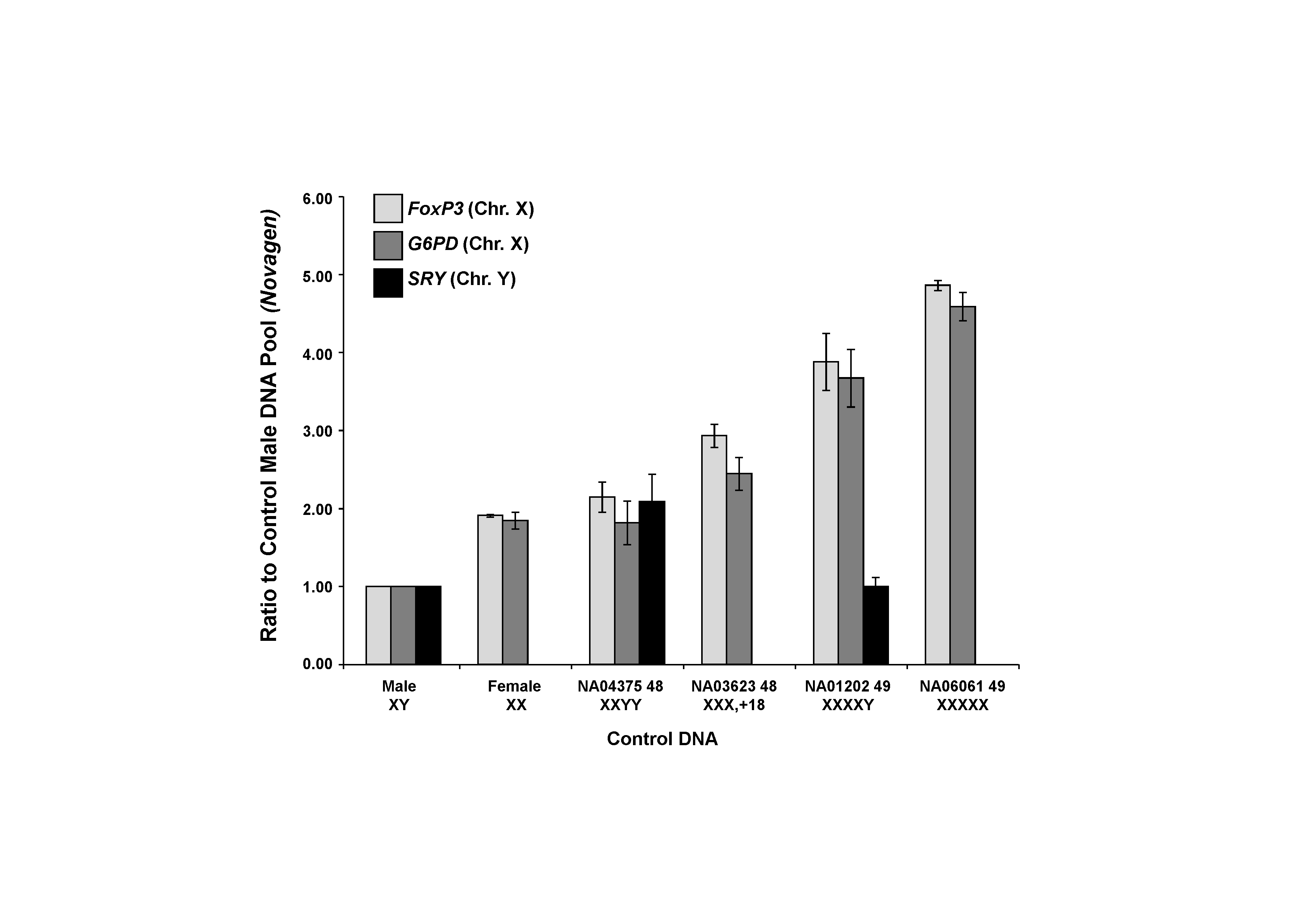

Supplement: Figure S1 — Model TaqMan qPCR assay. Conditions for TaqMan qPCR were optimized to precisely and reproducibly detect copy numbers from zero to five in control DNA samples with known copy numbers using genetic markers located on ChrX and ChrY and DNA from individuals or cell lines with various known numbers of chromosomes X and Y. X: one copy of chromosome X, Y: one copy of chromosome Y, FoxP3: gene present at chromosome X, G6PD: gene present at chromosome X, SRY: gene present at chromosome Y. (TIF) [file pone.0079790.s001.tif]
