# Supplementary material for: A Common Copy Number Variation (CNV) Polymorphism in the CNTNAP4 Gene: Association with Aging in Females
Source: PLoS One. 2013 Nov 6;8(11):e79790. doi: 10.1371/journal.pone.0079790 (PMC3819343; doi:10.1371/journal.pone.0079790)
Supplement: Table S1 — Investigated copy number variants and their harboring genes. Coordinates are provided for the assembly version NCBI Build 36.3 (UCSC hg 18). (DOC) [file pone.0079790.s002.doc]

| NCBI dbVar IDs* | Chromosome | Start | End | CNV type | Harboring gene |
| --- | --- | --- | --- | --- | --- |
| Esv16892(CNVR250.1) | 1 | 92,004,671 | 92,006,010 | deletion | *TGFBR3* |
| Esv19377(CNVR187.3) | 1 | 54,864,917 | 54,866,382 | insertion | *ACOT11* |
| Esv21297(CNVR1669.1) | 3 | 191,219,955 | 191,223,268 | deletion | *LEPREL1* |
| Esv17278(CNVR1591.1) | 3 | 157,574,746 | 157,576,258 | deletion | *KCNAB1* |
| Esv14949(CNVR1307.1) | 3 | 16,214,126 | 16,216,320 | deletion | *GALNTL2* |
| Esv11058(CNVR2149.1) | 4 | 166,222,892 | 166,224,339 | insertion | *TMEM192* |
| Esv25921(CNVR2052) | 4 | 116,148,170 | 116,151,343 | deletion | *NDST4* |
| Esv19453(CNVR2664.1) | 5 | 159,282,379 | 159,283,692 | insertion | *ADRA1B* |
| Esv25879(CNVR3107) | 6 | 152,431,681 | 152,433,972 | deletion | *ESR1* |
| Esv19098(CNVR3022.1) | 6 | 105,367,477 | 105,369,570 | deletion | *HACE1* |
| Esv19347(CNVR4427.1) | 9 | 106,636,036 | 106,637,657 | deletion | *ABCA1* |
| Esv18824(CNVR4644.1) | 10 | 18,881,143 | 18,902,7555 | deletion | *NSUN6* |
| Esv14002(CNVR5303.2) | 11 | 106,736,726 | 106,748,680 | deletion | *CWF19L2* |
| Esv20858(CNVR5589.1) | 12 | 70,643,163 | 70,644,244 | insertion | *TPH2* |
| Esv20888(CNVR6455.1) | 15 | 75,117,485 | 75,119,797 | deletion | - |
| Esv17566(CNVR6493.1) | 15 | 83,857,919 | 83,860,178 | deletion | AKAP13 |
| Esv16500(CNVR6524.1) | 15 | 92,687,427 | 92,693,333 | insertion | MCTP2 |
| Esv12669(CNVR6782.1) | 16 | 75,096,634 | 75,101,530 | deletion | CNTNAP4 |
| Esv17072(CNVR7370.1) | 18 | 62,370,731 | 62,372,179 | deletion | CDH19 |
| Esv12008(CNVR7791.1) | 20 | 14,383,333 | 14,385,206 | deletion | MACROD2 |

*Alternative IDs based on Conrad et al., 2010 [15] are provided in the brackets.
