# Supplementary material for: A Common Copy Number Variation (CNV) Polymorphism in the CNTNAP4 Gene: Association with Aging in Females
Source: PLoS One. 2013 Nov 6;8(11):e79790. doi: 10.1371/journal.pone.0079790 (PMC3819343; doi:10.1371/journal.pone.0079790)
Supplement: Table S2 — Basic health characteristics of seniors from PolSenior collection. (DOC) [file pone.0079790.s003.doc]

| Disease* | 65-75 years old | | 80-90 years old | |
| --- | --- | --- | --- | --- |
|  | F  n=278 | M  n= 316 | F  n=253 | M  n=271 |
| Cognitive impairment | n=31 (11) | n=53(17) | n=112 (44) | n=95(35) |
| Type 2 diabetes mellitus | n=57 (21) | n=44 (14) | n=46 (18) | n=39 (14) |
| Cardio-vascular diseases | n=68 (24) | n=100 (32) | n=55 (22) | n=81 (30) |
| Stroke | n=10 (4) | n=18 (6) | n=19 (8) | n=26 (10) |
| Chronic lung disease | n=30 (11) | n=53 (17) | n=29 (11) | n=60 (22) |
| Cancer | n=15 (5) | n=14 (4) | n=13 (5) | n=19 (7) |
| Healthy (no above mentioned pathologies) | n=107 (38) | n=121 (38) | n=60 (24) | n=74 (27) |

F: females, M: males, *: disease ever diagnosed, n: number of individuals (per cent).
