# Supplementary material for: A Common Copy Number Variation (CNV) Polymorphism in the CNTNAP4 Gene: Association with Aging in Females
Source: PLoS One. 2013 Nov 6;8(11):e79790. doi: 10.1371/journal.pone.0079790 (PMC3819343; doi:10.1371/journal.pone.0079790)
Supplement: Table S3 — Sequences for primers/probes used in the experimental quantification of the CNVR6782.1 in the CNTNAP4 gene by TaqMan qPCR assay. (DOC) [file pone.0079790.s004.doc]

| Gene | Primers | | Probe* |
| --- | --- | --- | --- |
| *CNTNAP4* CNVR6782.1 | Forward Reverse | 5’TGCAACACAAAGGGAGTTCCT3’  5’GCAGATAAGGGAGAGTGAGTGACA3’ | 5’CCACGATGGCATCTGGCTCTACGTC3’ |
| *FOXP3* chrX | Forward  Reverse | 5’CTCTGCCATTTAACCTCTTGCA3’  5’AAAATAGTTCTCCCCGAGGTTGA3’ | 5’CCTTTGGTGTGCAAGTTACTCCGCTTCTT3’ |
| *SRY* chrY | Forward  Reverse | 5’TCTGGGATTCTCTAGAGCCATCTT3’  5’GCGACCCATGAACGCATT3’ | 5’CGCCTCTGATCGCGAGACCACA3’ |

*All probes were labeled with 6-fluorescein amidite (6-FAM) as 5’ reporter dye and Black Hole Quencher (BHQ) as 3’ quencher dye.
